# Supplementary material for: Microplastics Environmental Effect and Risk Assessment on the Aquaculture Systems from South China
Source: Int J Environ Res Public Health. 2021 Feb 15;18(4):1869. doi: 10.3390/ijerph18041869 (PMC7918702; doi:10.3390/ijerph18041869)
Supplement: Supplementary file 1 [file ijerph-18-01869-s001.pdf]

Supplementary materials

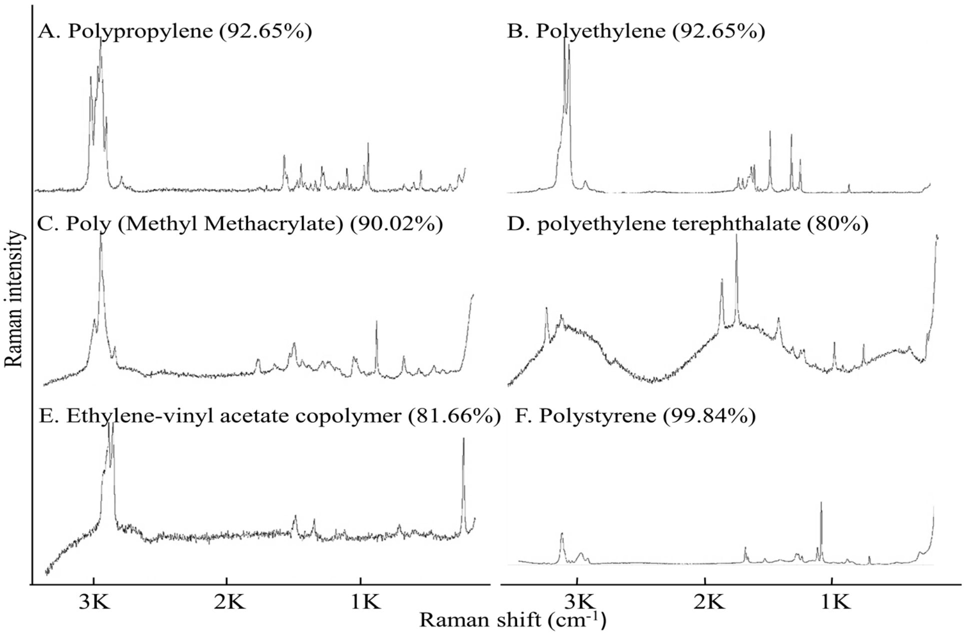

Figure S1. Composition of typical MPs identified by Raman spectroscopy.

Table S**1.** Body weight and body length information of commercial species collected from aquaculture farms in the Pearl River Estuary.

Species	Sample quantity	Length range (cm)	Average length (cm)	Weight range (g)	Average weight (g)
Oreochroms mossambcus	7	5.05 - 7.10	6.20 ± 0.71	1.78 - 6.14	4.12 ± 1.45
Micropterus salmoides	11	19.90 - 30.00	25.57 ± 2.81	156.82 - 526.24	328.86 ± 102.81
Penaeus vannamei	15	12.30 - 15.5	14.20 ± 1.12	9.64 - 22.10	16.09 ± 3.92
Macrobrachium rosenbergii	4	12 - 14.8	13.49 ± 1.19	11.2 - 16.98	14.13 ± 2.37

Table S**2.** The microplastic abundance of water and sediment in other studies compared with this study.

Sample type	Sampling site	Sampling method	Seperation method	Abundance (items/L)	Reference
Surface water					
	Yangtze River Estuary, China	screw pump	30% H_2O_2	0.16 ± 0.07	[59]
	Small-scale estuaries, Shanghai	stainless steel apparatus	30% H_2O_2	27.8 ± 11.81	[60]
	Charleston Harbor Estuary,USA	stainless steel and aluminum screen sampler	30% H_2O_2	3-36	[81]
	Three estuaries in Zhejiang, China	Teflon pump	5% formalin solution	0.10-4.10	[34]
Sediment					
	Changjiang Estuary, China	precleaned bottles	sodium chloride	12.1 ± 0.9 particles per 100 g of dry sediment	[30]
	Mediterranean Sea	5 mL metal-capped glass bijoux jar	Saturated 348 g NaCl/L solutin	182.66 ± 27.32 649.33 ± 184.02 items/kg	[67]
	River Thames, UK	Stainless steel scoop	1.7-1.8 kg/L $ZnCl_2$ solution	660 ± 77 items/kg	[66]

Table S**3.** The microplastic abundance of aquatic organisms in other studies compared with this study.

Species	Sampling site	Extraction method	Abundance (items/individual)	Reference
Fish				
6 marine fish	The Gulf Coast of Texas, US	_	0.5-1.4	[73]
5 fish species	Northeast Atlantic around Scotland	Dissection microscope	1.8 ± 1.7	[74]
5 marine fishes	the North and Baltic Sea	_	0.03 ± 0.18	[75]
Squalus acanthias	Adriatic Sea	30% H_2O_2	1.25 ± 0.5	[76]
Merlucius merluccius	Adriatic Sea	30% H_2O_2	1.33 ± 0.57	[76]
Mullus barbatus	Adriatic Sea	30% H_2O_2	1.57 ± 0.78	[76]
Chelidonichthys lucernus	Adriatic Sea	30% H_2O_2	1 ± 0	[76]
Sardina. pilchardus	Adriatic Sea	30% H_2O_2	1.78 ± 0.7	[76]
Scyliorhinus canicula	Cantabrian coast	NaOH	1.20 ± 0.45	[77]
Mullus barbatus	Spanish Coast	NaOH	1.75 ± 1.14	[77]
Konosirus punctatus	Xiangshan Bay	10% KOH 30% H_2O_2	2.1 ± 0.38	[24]
Larimichthys crocea	Xiangshan Bay	10% KOH 30% H_2O_2	1.8 ± 0.42	[24]
Oreochroms mossambcus	Pearl Rvier Estuary	10% KOH	6.14 ± 3.80	This study
Micropterus salmoides	Pearl Rvier Estuary	10% KOH	39.64 ± 23.38	This study
Crustaceans				
Parapenaeopsis. hardwickii	Xiangshan Bay	10% KOH 30% H_2O_2	0.95 ± 0.28	[24]
Penaeus vanname	Pearl Rvier Estuary	10% KOH	10.87 ± 4.94	This study
Macrobrachium rosenbergii	Pearl Rvier Estuary	10% KOH	9 ± 3.16	This study
